# Effect of Reactive Black 5 azo dye on soil processes related to C and N cycling

**DOI:** 10.7717/peerj.4802

**Published:** 2018-05-22

**Authors:** Khadeeja Rehman, Tanvir Shahzad, Amna Sahar, Sabir Hussain, Faisal Mahmood, Muhammad H. Siddique, Muhammad A. Siddique, Muhammad I. Rashid

**Affiliations:** 1 Environmental Sciences & Engineering, Government College University, Faisalabad, Pakistan; 2 Department of Food Engineering, National Institute of Food Science and Technology, University of Agriculture Faisalabad, Faisalabad, Pakistan; 3 Department of Bioinformatics and Biotechnology, Government College University, Faisalabad, Pakistan; 4 Center of Excellence in Environmental Sciences, King Abdul Aziz University, Jeddah, Saudi Arabia; 5 Department of Environmental Sciences, COMSATS Institute of Information Technology, Vehari Campus, Vehari, Pakistan

**Keywords:** Soil respiration, Litter amendment, Soil contamination, Soil microbial biomass, Azo dye contamination

## Abstract

Azo dyes are one of the largest classes of synthetic dyes being used in textile industries. It has been reported that 15–50% of these dyes find their way into wastewater that is often used for irrigation purpose in developing countries. The effect of azo dyes contamination on soil nitrogen (N) has been studied previously. However, how does the azo dye contamination affect soil carbon (C) cycling is unknown. Therefore, we assessed the effect of azo dye contamination (Reactive Black 5, 30 mg kg^−1^ dry soil), bacteria that decolorize this dye and dye + bacteria in the presence or absence of maize leaf litter on soil respiration, soil inorganic N and microbial biomass. We found that dye contamination did not induce any change in soil respiration, soil microbial biomass or soil inorganic N availability (*P* > 0.05). Litter evidently increased soil respiration. Our study concludes that the Reactive Black 5 azo dye (applied in low amount, i.e., 30 mg kg^−1^ dry soil) contamination did not modify organic matter decomposition, N mineralization and microbial biomass in a silty loam soil.

## Introduction

Azo dyes, which contain one or more than one -N=N- groups, constitute the largest class of synthetic dyes that are used in a wide range of commercial applications, i.e., textile, food, paper printing, cosmetics with textile industry as the largest consumer (O’Neill et al., 1999). It has been reported that 15–50 % of the applied azo dyes do not bind to the fabric during the dyeing process and are released into wastewater (McMullan et al., 2001). The concentration of the azo dyes in textile wastewaters vary from 10 to 250 mg L^−1^ (O’Neill et al., 1999). Presence of azo dyes in water bodies causes aesthetic problems and obstruct light penetration and oxygen transfer into water thereby affecting aquatic life (Sponza, 2006; Li et al., 2012; Zhang et al., 2012). Azo dyes and their degradation intermediates may also be mutagenic and carcinogenic for living organisms (Weisburger, 2002). In fact, the toxicity and ecological harm caused by organic dyes is much larger than that is caused by other organic pollutants such as PAHs and PCBs making them very important class of pollutants (Zhou, 2001).

Using the dye-containing textile wastewater for soil irrigation purposes around big cities is a common practice in developing countries (Ensink et al., 2004; Ensink, Van der Hoek & Amerasinghe, 2006; Faryal, Tahir & Hameed, 2007; Garg & Kaushik, 2008). Therefore, a large concentration of azo dyes can accumulate in surface soils particularly around textile processing units. For example, Zhou (2001) reported that on average 12.3–456.2 mg kg^−1^ soil of azo dyes were present in surface soils near dyeing and printing industry units. The dyes can stabilize in soil colloid within a few weeks and are retained in the soils for long term (Imran et al., 2015). In soils, they negatively affect germination rates as well as overall growth of plants (Cicek et al., 2012; Vafaei et al., 2012). There are a number of studies that have investigated soil processes to find the mechanistic basis for negative effect of azo dyes on plant growth and germination (Gupte, Keharia & Gupte, 2013; Imran et al., 2015; Mahmood et al., 2017; Shafqat et al., 2017).

For example, ${\mathrm{NH}}_{4}^{+}$-N oxidation potential, arginine ammonification rate and potential nitrification rates have been found to decrease after the soils were spiked with azo dyes (Topaç et al., 2009; Batool et al., 2015). Moreover, microorganisms involved in nitrogen (N) transformation events such as nitrobacter and ammonia-oxidizing bacteria as well as activity of N cycling enzyme urease have also been shown to decrease significantly in the presence of azo dyes (Oranusi & Ogugbue, 2002; Topaç et al., 2009; Batool et al., 2015). While soil N cycling in response to azo dye pollution has been investigated previously (Topaç et al., 2009; Batool et al., 2015), soil carbon (C) cycling which is closely coupled to N cycling and is primordially important for soil functioning has not been studied at all.

It has become a common practice to add organics to soils contaminated by hydrocarbons for their bioremediation (Lal, 2004; Gärdenäs et al., 2011; Shahzad et al., 2012). Organic amendments to soils immobilize hydrocarbon pollutants and reduce the negative effects on soil microbial pollutions and enzyme activities probably due to the role of organic matter in sorption of organic pollutants (Maliszewska-Kordybach & Smreczak, 2000; Tejada et al., 2008; Lee, Oh & Kim, 2008). How well an organic amendment to a soil contaminated with azo dyes improves the soil functions and alleviates its negative effects remains unknown.

Bioremediation of azo dyes in textile waste effluents in liquid media by bacteria has been widely studied (Khalid, Arshad & Crowley, 2008; Khalid et al., 2013; Hussain et al., 2013; Anwar et al., 2014; Najme et al., 2015). Several species of bacteria have been identified which decolorize azo dyes (Pandey, Singh & Iyengar, 2007; Hussain et al., 2013). Bacteria can even degrade intermediate products of decolorization such as aromatic amines with the help of enzymes like hydroxylase and oxygenase (Pandey, Singh & Iyengar, 2007), thus aiding in reducing lethal effects of azo dyes by formation of non-toxic metabolites. Despite this large number of studies reporting the potential of bacteria to transform azo dyes into non-toxic metabolites in liquid media, the evaluation of such bacteria to detoxify an azo dye in soil has rarely been assessed.

This study was designed to investigate the effect of an azo dye Reactive Black 5 (RB5) contamination on soil respiration, microbial biomass and net mineral N (ammonium & nitrate) in the presence or absence of litter and a bacterial species known to decolorize RB5 (Hussain et al., 2013).

## Materials & Methods

### Soil respiration

Soil was sampled from a non-contaminated irrigated agricultural field of experimental area of Ayub Agriculture Research Farm Faisalabad, that has been under wheat-fallow rotation for more than fifteen years. The upper 0–15 cm was sampled and sieved at 2 mm. Physico-chemical characteristics of the soil were determined (Table 1). Microcosms containing soil in sealed mason jars were prepared to measure soil C mineralization. Briefly, fifty grams (dry equivalent) of fresh soil of known water holding capacity (WHC, amount of water that a soil can hold at atmospheric pressure against gravity) and moisture content were put in a 100 ml beaker that was sealed in a 1 L mason jar. A test tube containing 40 ml 0.05 M NaOH was also placed inside jar to capture the CO_2_ evolved from the soil. Moreover, a test tube containing distilled water was also placed inside the jar to avoid the inside air from drying. Nine treatments of the study are given in Table 2.

Dye was mixed in ultra-pure water before being added to soil at the rate of 30 mg kg^−1^ soil. This rate of the azo dye was selected based on the average amount of it found in peri-urban soils of Faisalabad (study location) that are irrigated with textile wastewater (S Hussain, pers. comm., 2015). The strain RA20 (*Pseudomonas* sp.) was grown in MS medium (Hussain et al., 2013) and, after 24 h, the bacteria were harvested by centrifugation (6,000 rpm for 5 min), washed twice then re-suspended in water. This bacterial suspension was inoculated in the soil as per treatment plan. Senesced maize leaves were ground in a ball mill and added to relevant treatments at the rate of 1 g C kg^−1^ soil. Soil moisture content was maintained at 60% of the field capacity in all the treatments throughout the experimental duration of 29 days.

Soil respiration was measured by taking the NaOH traps out at regular intervals and concentration of CO_2_ evolved from soils was determined by a modified Isermeyer method (Isermeyer, 1952; Jaggi, 1976). Briefly, concentration of CO_2_ in NaOH was precipitated with 0.5 M BaCl_2_ followed by titration against 0.1 M HCl using phenolphthalein as indicator. At each gas sampling day, glass vials with fresh NaOH were placed and soil moisture content of soils were maintained at 60% of WHC by adding ultra-pure water when required. The lost water in soils was determined by weighing the soils packed in beakers.

**Table 1:** Physico-chemical properties of the soil used in the experiment.

Property	Value
Soil texture	Silt loam
Sand (%)	25
Silt (%)	55
Clay (%)	20
pH (1:2.5 soil to water)	8.32 ± 0.06
Electrical conductivity (µS cm^−1^)	1,151
Soil organic carbon (g kg^−1^ soil)	8.66 ± 0.43

**Table 2:** Details of the soil treatments used in the experiment.

Treatment name	Description/Dose
1. Control	Soil without any amendment.
2. Dye treatment	Soil spiked with Reactive Black 5 Dye (RB 5, 30 mg kg^−1^ soil).
3. Bacteria treatment	Soil inoculated with *Pseudomonas* sp. RA20 (Hussain et al., 2013).
4. Dye + bacteria treatment	Soil spiked with RB 5 (30 mg kg^−1^ soil) as well as inoculated with *Pseudomonas* sp. RA20.
5. Litter treatment	Soil amended with maize litter (1 g C kg^−1^ soil).
6. Dye + litter treatment	Soil amended with RB 5 (30 mg kg^−1^ soil) and maize litter (1 g C kg^−1^ soil).
7. Dye + litter + bacteria treatment	Soil amended with RB 5 (30 mg kg^−1^ soil) and maize litter (1 g C kg^−1^ soil) and inoculated with *Pseudomonas* sp. RA20.

### Soil variables

After 29 days of soil incubation, the experimental units were harvested for destructive sampling of soil and various soil variables were determined. Water extractable organic carbon (WEOC) of the soil was determined using the wet dichromate oxidation procedure described by Nelson & Sommers (1982). Briefly, 5 g of the incubated soil were shaken in 20 ml of ultra-pure water end over end for half an hour, centrifuged at 3,000 rpm and filtered. Four ml of the extract was taken and 1 ml of 1 M K_2_Cr_2_O_7_, 5 ml of concentrated H_2_SO_4_, and 2 drops of o-phenathroline monohydrate were added to it. The digests were then titrated against 0.033 M standardized ferrous ammonium sulphate.

Microbial biomass was determined using the fumigation-extraction method (Vance, Brookes & Jenkinson, 1987). Briefly, 10 g of soil was fumigated in the presence of fuming chloroform in an air-free desiccator for 24 h. Soils were then extracted with 40 ml of 0.5 M K_2_SO_4_ after shaking the mixture end over end for one hour. The extract was digested with potassium dichromate and concentrated sulphuric acid. The digests were then titrated against standardized ferrous ammonium sulphate after adding couple of drops of the indicator o-phenathroline monohydrate.

Soil nitrate N (${\mathrm{NO}}_{3}^{-}$-N) was determined colorimeterically by using salicylic acid nitration method (Cataldo et al., 1975) while soil ammonium N (${\mathrm{NH}}_{4}^{+}$-N) was determined by using Indophenol blue method (Keeney & Nelson, 1982).

### Statistical analyses

Since all the litter addition treatments showed markedly higher soil respiration rates, all the statistical analyses were performed by grouping litter amended treatments separate from those not amended with litter. However, control soil treatment was included in both types of tests. One-way ANOVA was used to test the effect of treatments on cumulative soil respiration, water extractable organic C, microbial biomass and soil ${\mathrm{NO}}_{3}^{-}$-N and ${\mathrm{NH}}_{4}^{+}$-N content. Least significance difference (LSD) was used to differentiate the treatments means when treatment effect was significant (i.e., *P* value < 0.05).

## Results

**Figure 1:**
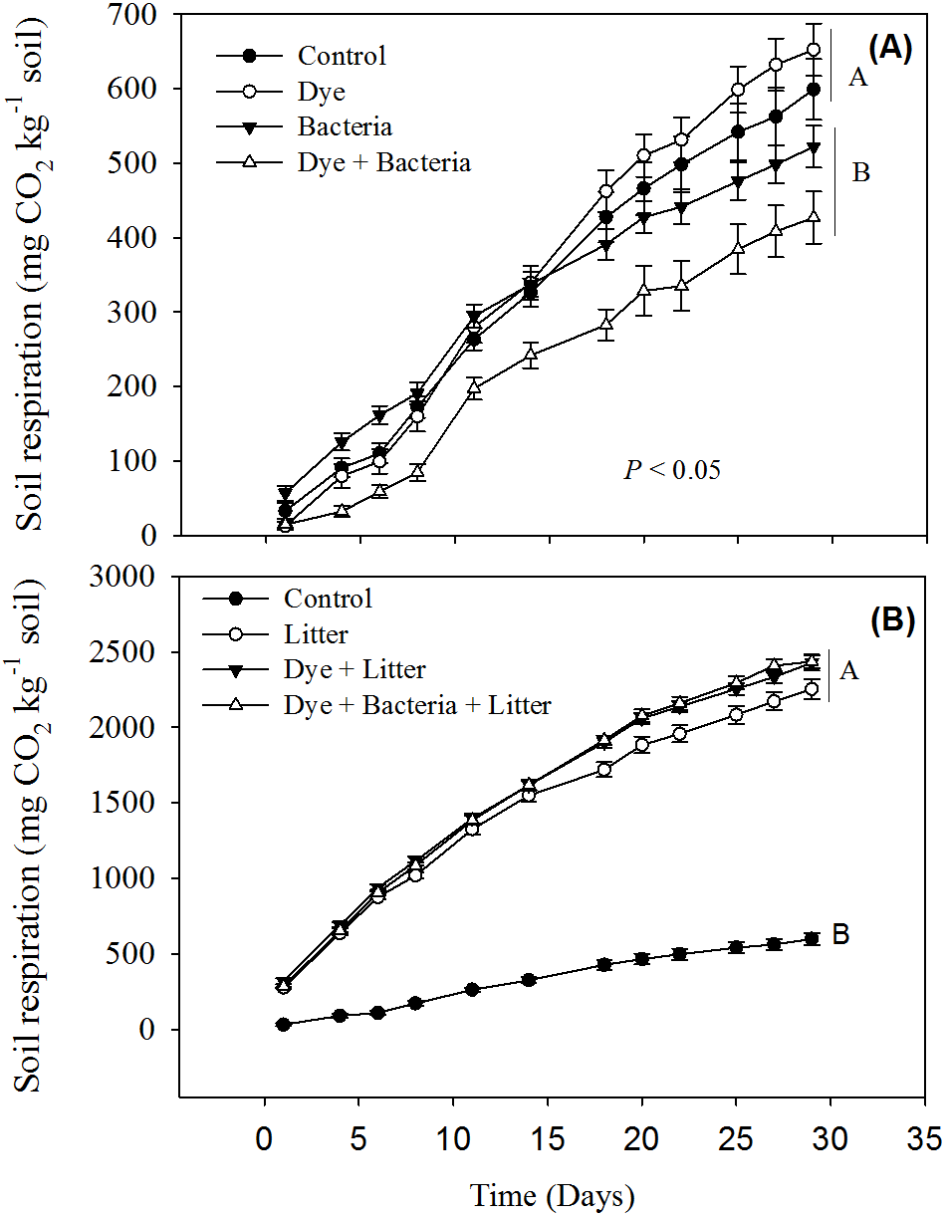
Soil respiration from different treatments. Soil respiration in response to dye, dye + bacteria and bacteria amendments in (A) the absence of and (B) presence of litter supply.

**Figure 2:**
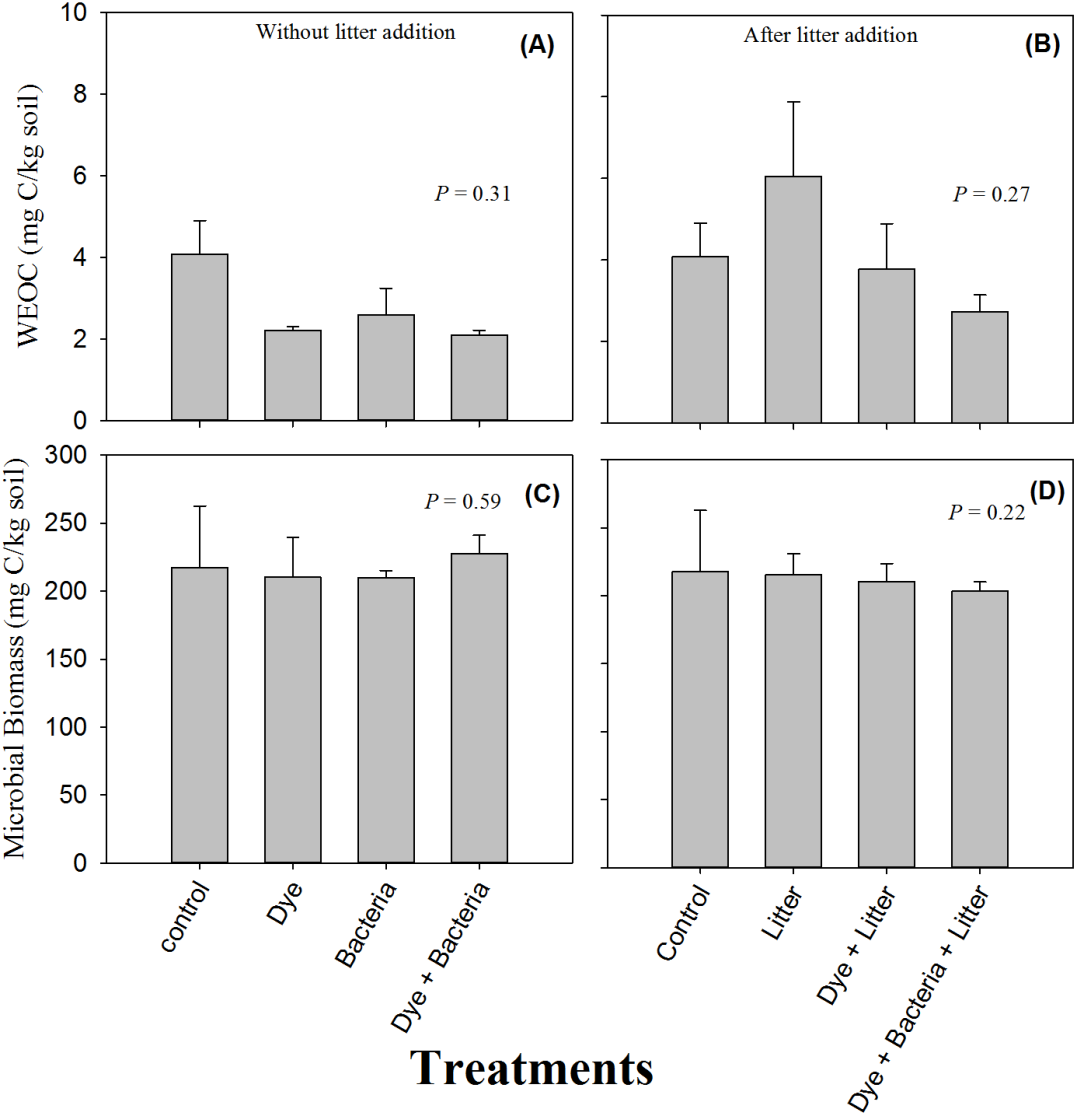
Water extractable organic C and microbial biomass. Water extractable organic carbon (C) and microbial biomass found in amended soils in (A & C) the absence and (B & D) the presence of litter supply respectively. Error bars are standard error of means.

Reactive Black 5 azo dye contamination did not have any effect on cumulative soil respiration (Fig. 1A). However, RB-5 decolorizing bacteria (*Pseudomonas* sp. RA20), when added alone or in presence of dye contamination, significantly reduced the soil respiration. All treatments involving litter amendment showed significantly much higher cumulative soil respiration than control (Fig. 2B). On average, all litter amended soils released 5x more C than the treatments excluding litter.

Availability of water extractable organic carbon (WEOC) or soluble C did not differ between control and treated soils (Figs. 2A & 2B, *P* > 0.05). Moreover, litter treatments did not differ from non-litter treatments in terms of soluble C (*P* > 0.05). Similarly, no treatment, whether involving litter amendment or not, induce any change in microbial biomass (Figs. 2C & 2D, *P* > 0.05).

**Figure 3:**
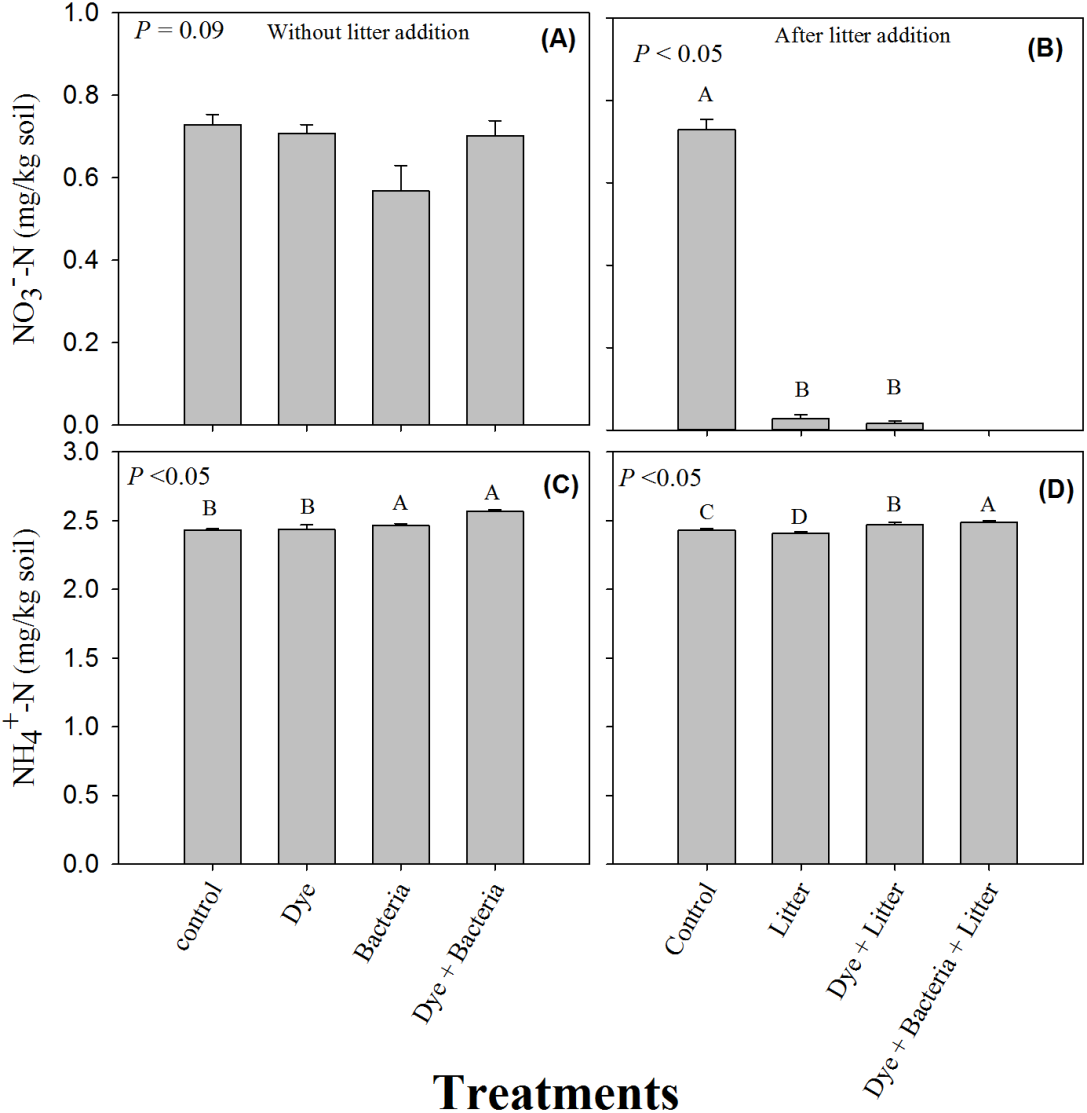
Mineral Nitrogen (ammonium and nitrate). Extractable nitrate and ammonium in amended soils in (A & C) the absence and (B & D) the presence of litter supply respectively. Error bars are standard error of means.

The soil ${\mathrm{NO}}_{3}^{-}$-N content remained unchanged under all the treatments where litter was not added (Fig. 3A, *P* > 0.05). However, litter addition alone or in combination significantly decreased soil ${\mathrm{NO}}_{3}^{-}$-N content (Fig. 3B, *P* < 0.05). The decrease was substantial and ${\mathrm{NO}}_{3}^{-}$-N content were undetectable in the dye + bacteria + litter treatment. Among treatments without litter addition, dye addition did not change soil ammonium content (Fig. 3C). However, soil ${\mathrm{NH}}_{4}^{+}$-N content were significantly higher in bacteria and dye + bacteria treatments. Among treatments with litter addition, highest ${\mathrm{NH}}_{4}^{+}$-N was found in dye + bacteria + litter treatment followed dye + litter and control treatments (Fig. 3D, *P* < 0.05). Lowest ${\mathrm{NH}}_{4}^{+}$-N was found in litter only treatment.

## Discussion

Our short-term study reveals that the azo dye RB-5 does not influence soil organic matter decomposition rates. Increase in soil respiration in response to litter amendment (Fig. 1B) is expected given that litter addition provides labile organic matter to energy-limited soil microorganisms (Sanaullah et al., 2010; Pascault et al., 2013; Kaneez-e Batool et al., 2016). The availability of labile carbon may stimulate the local microorganisms to accelerate the decomposition of extant organic matter—a phenomenon known as priming effect (Kuzyakov, Friedel & Stahr, 2000; Shahzad et al., 2015). In our study, the enormous amount of CO_2_ liberated (at least >2,200 mg CO_2_ kg^−1^ soil) in litter amended treatments compared to control soil (656 mg CO_2_ kg^−1^ soil) is a clear indication of priming effect. Dye contamination did not seem to suppress priming effect like overall soil respiration (Fig. 1B).

The decrease in soil respiration in presence of RB-5 decolorizing bacteria i.e., *Pseudomonas* sp. RA20 (Fig. 1A) was unexpected. In soils, there are several different functional groups of microorganisms which are in constant competition over resources and adapt and evolve in response to environmental changes (Fontaine, Mariotti & Abbadie, 2003; Shahzad et al., 2012; Perveen et al., 2014). We speculate that over the duration of our experiment, the introduced *Pseudomonas* sp. isolated and cultured from a stressful (industrial wastewater) environment, might have been stronger at competing for the resources than local microorganisms thereby decreasing their activity, i.e., soil respiration. We recommend future studies whereby competition of microorganisms isolated from stressful environments be studied with the microorganisms found in stress-free environments for determining the risk of inoculating such biological resources to local flora.

Azo dye did not induce any change in the availability of soil inorganic N in contradiction to previous studies (Topaç et al., 2009; Batool et al., 2015). Markedly reduced soil ${\mathrm{NO}}_{3}^{-}$-N in litter amended soils is an indication of microbial immobilization of soil nitrates to consume the available labile C to meet their stoichiometric demands of C and N (Moritsuka et al., 2004; Bengtson & Bengtsson, 2005; Shahzad et al., 2012).

Given that this is the first study which investigated soil C cycling under azo dye contamination, comparisons are impossible to make. However, the effect of azo dye contamination on N cycling which is closely linked with C cycling to the point that soil organic matter decomposition is positively related to inorganic N availability (Drake et al., 2011; Gärdenäs et al., 2011; Shahzad et al., 2015), has been extensively studied and may provide some comparisons. Azo dye contamination has been shown to result in reduction of inorganic N availability, lower rates of N transformation events, and reduced number of microorganisms and activity of enzymes involved in N (Topaç et al., 2009; Batool et al., 2015). Briefly, Topaç et al. (2009) found that RB5 (>20 mg kg^−1^ dry soil) and sulfonated azo dye (>8 mg kg^−1^ dry soil) decreased urease activity, arginine ammonification rate, nitrification potential and ammonium oxidizing bacteria numbers by 10–20% and 7–28%, respectively. The azo dye dose applied in their study is comparable to that in our study, i.e., 30 mg kg^−1^ dry soil. Perhaps the difference in soil texture could explain varying effect of the azo dye on soil processes. The soil we used was a silt loam while they used a sandy clay loam and differing stabilization of azo dyes might have resulted in varying effect on microbial processes (Imran et al., 2015). However, no effect on the microbial activity involved in organic matter decomposition found in our study warrant further research. In contrast, the doses of azo dyes in the study of Batool et al. (2015) where they recorded very high decreases in ammonium oxidation process and ammonia oxidizing bacteria numbers (>90%) were very high (400–1,600 mg kg^−1^ dry soil). Low dose of azo dye contamination in our study may explain our contradictory results vis-à-vis those of Batool et al. (2015).

No effect of azo dye contamination on soil microbial biomass was unexpected given that previous studies have repeatedly found reduced numbers of microorganisms in azo dye contaminated soils. For example, Imran et al. (2015) found reduced microbial biomass in the presence of all the three azo dyes including RB 5 that they used in their study. Perhaps the dose in our study was too low to have any deleterious effect on microorganisms because the dose used by Imran et al. (2015) was very high (160 mg kg^−1^ dry soil).

## Conclusion

In conclusion, our study shows that low levels of azo dye contamination (30 mg kg^−1^ dry soil) does not influence respiration or microbial biomass in a silt loam soil. However, we suggest that larger studies involving all the prominent azo dye types used in textile industries with a range of doses and different soil textures should be conducted to simultaneously determine the changes in closely linked C and N cycling processes. Moreover, we also propose that the effect of mixtures of azo dyes should be studied in future at different salinity levels mimicking the saline nature of textile wastewater commonly found.

##  Supplemental Information

10.7717/peerj.4802/supp-1 Supplemental Information 1 Nitrate, ammonium, MBC, WEOC

10.7717/peerj.4802/supp-2 Supplemental Information 2 Soil respiration

## Additional Information and Declarations

### Data Availability

The following information was supplied regarding data availability: The data are provided as Supplemental Files.
